# Sensitivity analysis for exploring the variability and parameter landscape in virtual patient cohorts of multi-vessel coronary artery disease

**DOI:** 10.1098/rsta.2024.0230

**Published:** 2025-04-02

**Authors:** Pjotr Hilhorst, Bregje van de Wouw, Karol Zajac, Marcel van ’t Veer, Pim Tonino, Frans van de Vosse, Wouter Huberts

**Affiliations:** ^1^ Department of Biomedical Engineering, Eindhoven University of Technology, Eindhoven, Noord-Brabant, The Netherlands; ^2^ Sano Centre for Computational Medicine, Extreme-Scale Data and Computing Team, Kraków, Poland; ^3^ Department of Cardiology, Catharina Hospital, Eindhoven, The Netherlands; ^4^ Computational Science Lab, Faculty of Science, Institute for Informatics, University of Amsterdam, Amsterdam, Noord-Holland, The Netherlands

**Keywords:** virtual patient cohorts, reduced-order modelling, one-dimensional pulse wave propagation model, fractional flow reserve, virtual cohort generators, correlated sensitivity analysis

## Abstract

Virtual patient cohorts (VPC) are crucial in *in silico* clinical trials, offering a promising, cost-effective and ethically advantageous alternative to real clinical randomized controlled trials to evaluate the safety and efficacy of clinical decision support tools and medical devices. This article focuses on the role of sensitivity analysis (SA) in evaluating a VPC created through a virtual cohort generator, which includes a one-dimensional pulse wave propagation model of the coronary circulation. Given the inherent limitations of clinical data, a synthetic VPC was generated that captured the global population variability of the fractional flow reserve distribution observed in the FAME study, a real-world randomized clinical trial. The synthetic VPC was created using random parameter variation and filtering with acceptance criteria, possibly inducing correlations between inputs. An SA methodology was employed that is able to account for correlations caused by acceptance criteria to explore the input–output relationship of the VPC and to explain its variability. The severity of the stenosis was found to be a key driver of the variability of the VPC. In general, the proposed SA approach, capable of handling correlated inputs, demonstrates an effective method for evaluating VPCs, providing a robust framework for *in silico* clinical trial applications.

This article is part of the theme issue ‘Uncertainty quantification for healthcare and biological systems (Part 2)’.

## 1. Introduction

The development and clinical introduction of new intervention protocols, tools to support intervention planning and innovative medical devices require an extensive and costly preclinical workflow. This includes, among others, bench testing and animal trials to assess their safety and effectiveness [1,2]. Aside from the extensive preclinical path, there is also a need for human randomized controlled trials (RCTs) to demonstrate efficacy. These RCTs are often very costly, requiring many patients, and therefore take up a vast amount of time and resources [3]. Further compounding challenges include notable disparities in the representation of diverse patient demographics within clinical trials. An overrepresentation of certain demographic groups leads to significant imbalances in clinical data, undermining the generalizability of research outcomes [4]. Additionally, identifying the underlying reasons for failure often presents considerable difficulties in instances where an RCT is classified as unsuccessful.

In this context, *in silico* clinical trials (ISCTs) emerge as a cost-effective, time-efficient and ethically advantageous approach to evaluate the safety and efficacy of clinical decision support tools and medical devices [5,6]. ISCTs potentially have numerous advantages, such as minimizing the need for animal testing, adjusting the size of RCTs, enhancing trial design and generalizability through improved inclusion–exclusion criteria and broadening the diversity of patients evaluated for overall safety and effectiveness compared with traditional RCTs. An ISCT is defined as a computational study that assesses a medical intervention using a cohort of computational patient models, i.e. a virtual patient cohort (VPC) [6].

One commonly applied method for generating VPCs involves creating synthetic virtual patients based on mathematical models that can realistically mimic the physiology of real patients, both before and after an intervention. The input parameters of this model are then simultaneously varied within their marginal distributions, defined by available population information, such as measurements, literature or common acceptance, to generate new members of the VPC [7]. Input sets leading to non-realistic physiological model responses are filtered out based on user-defined acceptance criteria. The physiological model, the inputs and the filtering strategy together form a virtual cohort generator (VCG), whereas the filtered inputs, generated outputs and the model represent a synthetic VPC. This method is advantageous due to its simplicity and the ability to build VPCs by leveraging *a priori* physiological knowledge conceptualized in the model. This is particularly beneficial given the inherent sparsity of real patient data and potential ethical constraints that can arise through the use of clinical data, which could hinder its practical application in a clinical setting [7,8]. However, a drawback is the uncertainty regarding the actual occurrence of the generated virtual patients in the real population. This leads to the generated VPC being strictly evaluated and filtered against population data to ensure only those within physiological bounds are included in the VPC [7]. This input-variation filter approach is deemed to be the most practical approach for generating a VPC for ISCTs. However, it does require an extensive validation process of the filtering technique used, the employed physiological model and the credibility of the resulting VPC.

Variance-based sensitivity analysis (SA) emerges as a critical component in the process of assessing model credibility and validating a VPC [6,9]. It is an important tool for quantifying the variance of the population distribution and understanding the structure of the VPC, the input landscape and the relationship between input and output. This is among others relevant when evaluating the VCG to gain insight into the key drivers that result in a certain population distribution within the VPC.

In this article, we demonstrate the utility of SA in evaluating a VPC, leveraging a one-dimensional pulse wave propagation model of the coronary circulation. A pulse wave propagation model was selected because these models have proven effective in simulating the propagation of pressure and flow waves throughout the cardiovascular system [10–12]. This capability of the pulse wave propagation model ensures that the inherent *a priori* physiological characteristics needed for our VCG are well represented, thereby justifying our approach. This model, developed as part of a VCG, showcases, on a population level, the power of ISCTs by comparing the computed fractional flow reserve (FFR) values in this study with the FFR measurements of a real-world RCT: the FAME study [13]. In 2009, the FAME study established the usefulness of fractional flow reserve as a metric for assessing the severity of coronary artery disease in multi-vessel disease, significantly reducing adverse events in a one-year follow-up [13]. Fractional flow reserve is an index of the physiological significance of coronary artery disease and is defined as the ratio of maximal blood flow in a diseased coronary artery compared with that in a healthy coronary artery [13]. FFR is measured, during hyperaemia, by dividing the pressure distal to the stenosis by the pressure proximal to the stenosis, typically using the aortic pressure as the reference for the latter. The resulting FFR value is calculated as the moving average over three consecutive heartbeats. These measurements are performed invasively using a pressure-guiding catheter. An FFR value of 0.8 or less is assumed to indicate an ischaemia-causing coronary stenosis [14]. Similar to numerous RCTs, the dataset acquired from the FAME study exhibits sparsity. Due to it being a clinical study, the study lacks the necessary completeness in information for model development and validation, thus advocating for the creation of a synthetic VPC through the process of parameter variations with acceptance criteria.

However, due to the practice of filtering samples from the virtually created population, there is a high probability that using the input-variation filter approach introduces dependencies into the input parameter distributions of the model. Furthermore, as shown before [15], the reliability of the SA metrics hinges on our precise identification and management of correlations between inputs. Additionally, many state-of-the-art global SA methods disregard correlations due to their high computational costs and the fact that the correlations are often unquantified [16]. These correlations can significantly impact the calculated sensitivity indices, potentially affecting conclusions regarding the model structure, the input–output relationships and the key drivers of the variability within the VPC.

Therefore, the aim of this study is to use a variance-based SA methodology, which is able to consider these possibly filter-induced correlations, to explore the input–output relations and the parameter landscape of a VPC that is built using the input-variation filter approach and leads to a population variability and physiological envelope of synthetic coronary artery disease patients. This careful examination aids in pinpointing the variables vital for enhancing the VCG’s accuracy and helps validate the resulting VPC, thus improving its reliability in determining the FFR for ISCTs. Additionally, by leveraging SA in this manner, we are, in turn, able to improve and/or evaluate the VPC, ensuring that it accurately represents the patient population in question and that the insights gained from using this VPC in an ISCT are a realistic reflection of the real world.

We first present our methodology in §2. Afterwards, to enhance clarity and focus, the study results are presented systematically across three different sections. Section 3 covers the generation of the VPC. Section 4 focuses on the construction of surrogate models to facilitate efficient SA. Section 5 addresses SA to explore the variability and parameter landscape of the generated virtual patient cohort.

## 2. Methods

First, we will give an overview of the one-dimensional pulse wave propagation model, which serves as the basis of our VCG. Then, the VPC is generated through the input-variation and filter approach, which completes the VCG, and the resulting VPC is compared to the literature and the FAME study in terms of population distribution. Furthermore, we will elaborate on how we identify the potential correlation structure of the resulting synthetic virtual patient cohort.

Following this, we will shortly outline our surrogate model-based SA to evaluate the impact of input parameters on model outputs, provide insight into the parameter landscape and quantify the population variability. Surrogate model-based SA encompasses two primary steps: the development and validation of surrogate models that approximate the behaviour of the full-order model and the execution of correlated variance-based SA [15].

### (a) One-dimensional pulse wave propagation model

**Figure 1.**
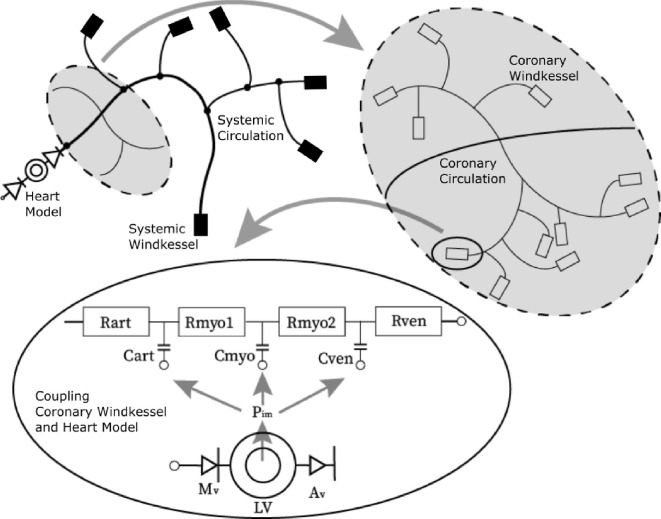
The one-dimensional pulse wave propagation model consisting of a truncated part of the systemic circulation, the coronary circulation and lumped models serving as boundary conditions.

The one-dimensional pulse wave propagation model is a multi-scale model consisting of one-dimensional line elements and different types of lumped elements. A schematic overview of the model and its various components can be seen in figure 1. The model consists of the systemic and coronary circulation, modelled as one-dimensional line elements. The peripheral vasculature at the truncated arteries is modelled using three-element Windkessels (systemic Windkessel), while specific lumped models (coronary Windkessel) are used for the coronary microcirculation. The stenotic lesions are modelled using nonlinear lumped resistances. The heart is modelled using a zero-dimensional one-fibre heart model, which serves as an inflow boundary condition and is coupled to the coronary microcirculation to simulate the effects of cardiac contraction on the coronary arteries. A full overview of the fundamental equations and computational techniques used to simulate coronary haemodynamics is provided in electronic supplementary material, appendix A.

#### (i) Topology

The systemic artery topology is adopted from the ADAN56 model [17] and has been reduced in a way that the haemodynamics is minimally affected by the reduction (see electronic supplementary material, appendix C). Additionally, the LIMA has been added to allow for the simulation of possible coronary artery bypass graft treatments in the future (see electronic supplementary material, appendix B). The resulting systemic artery topology can be found in table 1. The wall thickness of the systemic arteries was determined in accordance with Boileau *et al*. [18].

**Table 1.** Topology of the reduced systemic arterial circulation adopted from the ADAN56 model [17].

no.	length (cm)	radius proximal (cm)	radius distal (cm)	artery name
1	7.441	1.595	1.295	aortic arch I
2	4.735	0.673	0.616	brachiocephalic trunk
3	0.960	1.295	1.257	aortic arch II
4	12.132	0.448	0.333	left common carotid
5	0.698	1.257	1.228	aortic arch III
6	4.938	0.490	0.348	left subclavian I
7	4.306	1.228	1.055	aortic arch IV
8	20.415	0.134	0.134	left vertebral
9	2.056	0.348	0.289	left subclavian II
10	2.056	0.289	0.230	left subclavian III
11	15.000	0.100	0.100	LIMA

**Figure 2.**
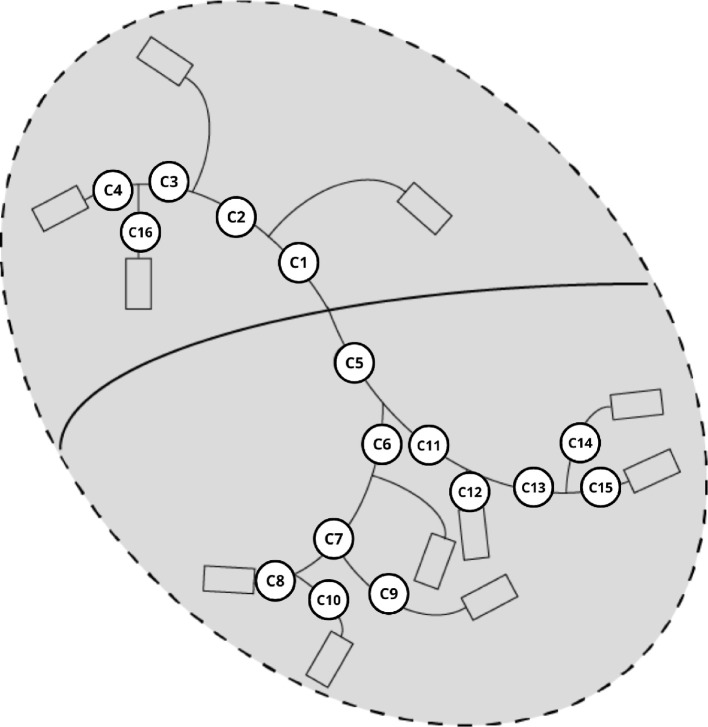
Schematic representation of the coronary artery topology including the segment numbers associated with them based on Kaiser *et al*. [19]. The rectangles at the distal side represent the coronary microcirculatory elements.

As for the coronary artery topology, in figure 2, we can see the schematic geometry of the coronary segments, representing the three main epicardial arteries. This geometry is based on the idealized geometry proposed by van der Horst *et al*. [20] but adjusted for the FAME study [13], where the segments are numbered following the methodology by Kaiser *et al*. [19].

Both the left main coronary artery (LM) and the right coronary artery (RCA) are set to originate 10 mm distal from the aortic valve [21]. The LM, being 2 cm, bifurcates into the left anterior descending (LAD) and circumflex (LCx) arteries. The LAD is 10 cm in total length and features three side branches representing the diagonal and septal side branches. The LCx is 8 cm in length and has two side branches, the marginal and posterior lateral branches. Similarly, the RCA has the same geometry as the LAD, measuring 10 cm long and having three side branches. A radius of 1 mm is prescribed for all coronary side branches [20], and the ratio of the mother and daughter vessels at each bifurcation is calculated using Murray’s law [22]. It is also assumed that the wall thickness of all coronary vessels equals 10% of the vessel’s radius [20].

### (b) Virtual patient cohort generation and verification

#### (i) Anatomical model selection

**Figure 3.**
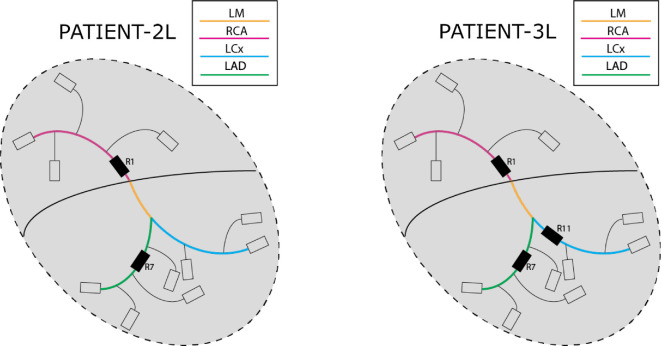
Schematic representation of two types of FAME study patients and their topologies with their stenoses located in the three main epicardial arteries.

The VPC will be generated using two stenotic anatomical models representing typical patient types with multi-vessel disease, capturing the key variations in lesion count relevant to the FAME study [13]. All patients in the FAME study suffered from multi-vessel disease, and here we focus on the subgroup with multiple lesions in the main coronary arteries (LAD, LCx and RCA). Within the FFR-guided group of the FAME study, 213/509 patients had stenotic lesions only within the main coronary arteries. To mimic these patients, we opted for the following two patient types: patient-2L has two stenotic lesions located in segment C1 (RCA) and segment C7 (LAD). Patient-3L has three stenotic lesions: one in segment C1 (RCA), one in segment C7 (LAD) and one in segment C11 (LCx). The geometries of both patient types can be seen in figure 3, respectively.

#### (ii) Input parameter variation and simulation

After demonstrating that the one-dimensional pulse wave propagation model can accurately simulate generic trends of coronary physiology (see electronic supplementary material, appendix A), it was utilized in a VCG. The input parameters of the one-dimensional pulse wave propagation model were quasi-randomly varied within their marginal distributions through uniform sampling, conducted in Python 3.9 using the function random.uniform() [23]. Afterwards, simulations were conducted with those specific input sets.

The model input parameters were varied as shown in table 2, including their respective ranges. Assuming uniform marginals, these ranges were based on ranges found in literature: volume fraction, Clay *et al*. [24]; $\sigma_{f0}$ , $c_{f}$ , $\sigma_{r0}$ , $c_{r}$ , Nikolic *et al*. [25]; $\sigma_{a0}$ , $c_{a}$ , de Tombe & ter Keurs [26]; $l_{s0}$ , $v_{0}$ , Janssen & Hunter [27], E: Awad *et al*. [28]; $P_{vo}$ , Tansey *et al*. [29]; η , Nader *et al*. [30]; ρ , Vitello *et al*. [31]; μ , Karimi *et al*. [32]; HF , Avram *et al*. [33]; $P_{mean}$ , $q_{tot}$ , Ramanathan & Skinner [34]; Stenosis percentage, Heinen *et al*. [35], Ramanathan & Skinner [34]; $f_{hyperemia}$ , Gould *et al*. [36]; $P_{pul\;ven}$ , Marks *et al*. [37]. For all other parameters, their ranges were chosen heuristically as fractions of their original values.

**Table 2.** Input parameters for the sensitivity analysis including their respective ranges.

parameter	minimum	maximum	unit	description
volume fraction	20	40	(%)	percentage of $V_{w}$ to determine $V_{lv0}$
$\sigma_{f0}$	$0.8\times 10^{3}$	$1.0\times 10^{3}$	(Pa)	scaling parameter fibre
$c_{f}$	11.5	12.5	(-)	curvature parameter fibre
$\sigma_{r0}$	$0.15\times 10^{3}$	$0.25\times 10^{3}$	(Pa)	curvature parameter radial
$c_{r}$	8.5	9.5	(-)	curvature parameter radial
$\sigma_{a0}$	$90\times 10^{3}$	$250\times 10^{3}$	(Pa)	scaling parameter active stress
$c_{a}$	$1\times 10^{6}$	$2\times 10^{6}$	(-)	curvature parameter active stress
$l_{s0}$	$1.9\times 10^{-6}$	$2.2\times 10^{-6}$	(m)	sarcomere length at reference state
$v_{0}$	$0.96\times 10^{-5}$	$1\times 10^{-5}$	(m s^−1^)	sarcomere shortening velocity
E	0.4	0.8	(MPa)	Young’s modulus
$P_{vo}$	0	1067	(Pa)	average venous outlet pressure
η	$3.5\times 10^{-3}$	$5.5\times 10^{-3}$	(Pa)	dynamic viscosity
ρ	$1.04\times 10^{3}$	$1.06\times 10^{3}$	(kg m^−3^)	blood density
μ	0.4	0.6	(-)	Poisson ratio
HF	60	70	b.p.m.	heart frequency/rate
$q_{tot}$	200	300	ml min^−1^	total flow to the coronary arteries
compliance scale	1.0	2.0	(-)	systemic Windkessel compliance scaling factor
resistance scale	0.71	1.43	(-)	systemic Windkessel peripheral resistance scaling factor
$R_{art\;frac}$	5/27	9/27	(-)	arterial resistance factor
$R_{myo1,2\;frac}$	7/27	11/27	(-)	myocardial resistance factor
$R_{ven\;frac}$	0	4/27	(-)	venous resistance factor
$C_{art}$	0.02	0.38	mm^3^Pa^−1^	arterial compliance
$C_{myo}$	0.053	1.007	mm^3^Pa^−1^	myocardial compliance
$C_{ven}$	0.065	1.235	mm^3^Pa^−1^	venous compliance
$P_{im_{frac}}$	1/2	1	(-)	intramyocardial pressure fraction
stenosis percentage			(%)	severity percentage of the stenosis
LCx	30	70		
LAD	30	70		
RCA	30	70		
stenosis length			(mm)	length of the stenosis
LCx	3.0	17.0		
LAD	3.0	37.0		
RCA	3.0	17.0		
position stenosis	0.1	0.9	(-)	position of a stenosis in a segment
$P_{mean}$	60	140	(mmHg)	mean arterial pressure
$f_{hyperemia}$	2	5	(-)	hyperaemia factor
$P_{pul\;ven}$	267	1600	(Pa)	pulmonary venous pressure

The lengths of the stenoses, depicted in figure 3, were selected to vary from three elements of a segment to all elements, with the exception of the first and last elements. This latter criterion is employed to preclude the possibility of a stenosis occurring on a bifurcation since the current zero-dimensional stenosis element is not yet capable of dealing with a pressure drop over a stenosis located at a bifurcation. Similarly, in segment C7, the stenosis was positioned solely in the proximal portion, before the bifurcation that leads to segment C9. This proximal section of segment C7 is twice as long as segments C1 and C11. Consequently, a larger range was applied to the stenosis length within the LAD. Due to having two patient types, the input space for patient-2L contains 34 parameters due to stenoses being present only in the LAD and RCA, and 36 parameters for patient-3L with stenosis present in all three main coronary arteries.

In total, 5000 input sets were generated per patient type and used to run 5000 simulations with the one-dimensional pulse wave propagation model for each of the two patient types. Since the heart frequency varied, the time step size and the time of a cardiac cycle per sample differed, but it was ensured each cardiac cycle was normalized over the sample-specific cycle time; hence, each cardiac cycle was distributed over 1000 data points.

Within the ambit of 5000 simulations conducted for patient-2L and patient-3L, a total of 3934 and 3946 simulations, respectively, ran successfully, whereas the remainder terminated prematurely. The premature termination of simulations can occur due to instabilities arising from implausible input parameter combinations, which was also the case here, where numerical instabilities arose within the one-fibre heart model. To ensure the quality of the VPC, pressure signals from completed simulations were also filtered to remove any strong oscillations that could also result from certain input parameter combinations and numerical solver aspects, such as timestep size and element size. A second-order Butterworth filter with a sampling and cut-off frequency of 1000 and 100 Hz, respectively, was selected on the basis of its optimal flat frequency response within the passband. This ensures minimal distortion of the low-frequency components. The filtering was done in MATLAB2023a using the filtfilt function, which applies the filter in both forward and reverse directions to eliminate phase shifts, resulting in a zero-phase distortion filtered signal. The root mean square (RMS) value was calculated to assess the difference between the original and filtered pressure signals, thus quantifying the extent to which the high-frequency signals had been removed. All waveforms with an RMS>4[Pa] were filtered out since they were identified to contain significantly high-frequency components. This ensured our VPC did not contain strong numerical oscillations, which might interfere with the training of the surrogate models in §2.

#### (iii) Filtering the virtually created population within physiological bounds

A subset of outputs of these VPCs, the coronary flow, the aortic pressure and the cardiac output were filtered to remain within physiological bounds. The diastolic aortic pressure should remain above 50 mmHg, and the systolic aortic pressure should remain below 150 mmHg [38]. Since hyperaemia is applied, the blood volume flow should remain between 300 and 900 ml min^−1^ within the left main coronary artery [39,40]. Cardiac output values outside the range of 3.5−6 l min^−1^ were also filtered out, representing the typical resting cardiac output range. The chosen lower range is slightly lower than the physiological lower limit of 4 l min^−1^ [41]. However, due to the reduction of the systemic geometry, a relatively lower flow was needed to attain physiological pressure signals, which is our main interest with regard to FFR prediction.

#### (iv) Virtual patient cohort verification

After the creation of the VPC through filtering based on the three physiological bounds described in §2, the resulting synthetic VPC was then verified by population-level comparison to literature and the data from the FAME study. For comparison with the FAME study, we selected, from the subset of patients with multi-vessel disease and stenotic lesions located exclusively in the three main coronary arteries (LAD, LCx and RCA), an additional subgroup of 167 patients. In this subgroup, the stenotic lesions were neither located near a bifurcation nor present in two consecutive segments. Lastly, we conducted a Kolmogorov–Smirnov (KS) test to statistically assess whether the null hypothesis—that the two samples originate from the same distribution—can be retained.

#### (v) Correlation structure derivation of the virtual patient cohort

Additionally, for VPC generation, since the results were filtered based on physiological constraints, there is a chance that correlations might have been introduced into the input parameter sets of the VPC.

To ensure that the correlation matrices of the VPCs were accurately identified, a Gaussian copula was employed using the MATLAB copulafit function [42]. The copula approach is used because it is more generally applicable, also in cases when the marginal distributions are not Gaussian. The Gaussian copula separates the correlation structure from the marginal distributions. The resulting correlation matrices can be found in electronic supplementary material, appendix E. The analysis of the resulting correlation matrices for both patient types reveals a mix of weak and moderate correlations, with only a few pairs demonstrating notable relationships. Most correlations are below ± 0.1, indicating weak or negligible linear relationships among the parameters. The strongest correlations are found for some parameters of the one-fibre heart model. These correlations are between volume fraction and pulmonary venous pressure ( ρ= −0.25/−0.22 for patient 2L/3L), sarcomere length at reference state and curvature parameter active stress ( ρ= −0.35/−0.36), sarcomere length at reference state and scaling parameter active stress ( ρ= −0.34/−0.36) and scaling parameter active stress and curvature parameter active stress ( ρ= −0.44/0.43).

### (c) Surrogate model construction for efficient sensitivity analysis

To efficiently conduct the variance-based SA on the virtual patient cohort, we utilized the surrogate model-based SA method, as detailed in [15]. A surrogate model $S:{\mathbb{R}}^{d}\to {\mathbb{R}}^{,}$ of the original one-dimensional pulse wave propagation model is generated using a method known as the vectorial kernel orthogonal greedy algorithm (VKOGA), resulting in a surrogate model of the form


(2.1) $$\begin{array}{lcr} & S(x):=\sum_{j=1}^{n}\alpha_{j}K(x,x_{j}),\;x\in {\mathbb{R}}^{d}. & \end{array}$$

This kernel-based method was introduced by Santin & Haasdonk [43]. VKOGA enables the creation of a surrogate model using a finite set of input and output variables. More information about this method can be found in [43]. The output of interest for the SA was chosen to be the FFR for each stenosis located within the patient types, resulting in a total of five surrogate models.

#### (i) Training and test set creation

Training sets need to be defined to train surrogate models of the one-dimensional pulse wave propagation model. The original VPCs, after filtering out the numerical unstable solutions and the waveforms containing oscillations and before filtering based on physiological bounds, were used as training and test sets to ensure that the oscillations would not interfere with the training process. There is no necessity to eliminate non-physiological results because the surrogate models are solely focused on understanding the relationship between the model inputs and outputs of the original model. The original VPC dataset was split, resulting in a training set of at least 2500 simulations, and the remaining simulations were added to the test set, leaving 820 and 844 simulations for patient-2L and patient-3L, respectively. Eventually, five surrogate models have been trained in total for each FFR value belonging to a stenosis of a certain coronary artery. Possible interactions between stenotic lesions are still considered since each surrogate demonstrates the FFR of one lesion in a patient with multiple stenotic lesions.

#### (ii) Training of the surrogate models

The surrogate models were trained on the training dataset while using a Matern k2 radial basis function (RBF) kernel ( $RBF(\epsilon ,r)=\exp(-\epsilon r)(3+3\epsilon r+(\epsilon r{)}^{2}$ with ϵ being the shape parameter and r the radial variable) and the f -greedy algorithm [43]. A Matern k2 RBF kernel was chosen over a Gaussian RBF kernel, as it was assumed that data were not infinitely smooth. f -Greedy was chosen to ensure the model output was incorporated into the training of the surrogates to ensure supervised learning. We refer the reader to [43] for more information about greedy algorithms. All other settings, i.e. for hyperparameter optimization, were adopted from [15].

#### (iii) Surrogate model performance

The output range of the training set was analysed to show the spread of FFR values obtained for each stenotic lesion and patient type used in the training of the surrogate models. This visualization helps to clarify the variability in FFR values within the training data, providing a clearer view of the range of the training data used in the surrogate construction process.

The performance of the candidate surrogate models was quantified by computing the root mean square error between the surrogate prediction $S(x_{i})$ and the test set result $y_{i}$ :


(2.2) $$\epsilon_{RMSE}:=\sqrt{\frac{1}{n}\sum_{i=1}^{n}∥S(x_{i})-y_{i}{∥}^{2}},$$

with n being the number of elements of x , S the surrogate model that is being evaluated and ∥...∥ the $\ell^{2}$ -norm. The normalized root mean square error ( $\epsilon_{nRMSE}$ ) was computed as well to simplify the comparison by dividing the root mean square error by the mean FFR of the test set. Additionally, Bland–Altman plots were generated to assess the agreement between the surrogate predictions and the test set results, enabling the identification of any systematic bias and the determination of the limits of agreement [44].

### (d) Exploring the variability and parameter landscape of the generated virtual patient cohort

The same variance-based surrogate model-based SA methodology was employed, as described in [15], to explore the variability and parameter landscape of the generated virtual patient cohort.

#### (i) Definitions of the sensitivity indices

Performing variance-based correlated SA results in the computation of the total correlated ( $S_{TC}$ ) and total uncorrelated ( $S_{TU}$ ) sensitivity indices [16]:


(2.3) $$\begin{array}{lcr} & S_{i}^{TC}=\frac{V(E(Y|X_{i}))}{V(Y)}\;\text{and}\;S_{i}^{TU}=\frac{E(V(Y|X_{-i}))}{V(Y)}. & \end{array}$$

Here $X_{i}$ is a model input, Y represents the model output, $E(Y|X_{i})$ is the conditional expected value when $X_{i}$ is known, $E(Y|X_{-i})$ is the conditional expected value when all other inputs but $X_{i}$ are known and V(Y) is the output variance. Note that the operators are similar to well-known Sobol indices ( $S_{main}$ and $S_{total}$ ) [45]. However, the assumption that the joined probability density function can be written as a product of the marginal distributions no longer holds because inputs are no longer statistically independent. This causes a change in the integrand definitions of both equations and their interpretation [16].

In the context of virtual cohort generation, the total correlated sensitivity index ( $S^{TC}$ ) can be decomposed into the correlated ( $S_{C}$ ) and uncorrelated ( $S_{U}$ ) components. $S_{C}$ represents the correlated variance contribution of the input parameter with other input parameters and the contribution by interactions associated with those correlations. $S_{U}$ is the independent variance contribution of the input parameter itself. As for the total uncorrelated sensitivity index $S^{TU}$ , it represents the contribution of a certain input parameter to the variability within the VPC without the variability associated with the correlation of that input parameter with other parameters. Similarly, $S_{TU}$ can be decomposed into the uncorrelated ( $S_{U}$ ) component and an additional component, explaining the variance contribution due to interactions between the parameter itself and others ( $S_{IU}$ ) and without correlations [16].

The individual components $S_{C}$ (correlated), $S_{U}$ (uncorrelated) and $S_{IU}$ (interaction) enable a detailed decomposition of the total variance contributed by each input parameter. By separating the total correlated and uncorrelated sensitivity indices into these components, we can directly associate the variance contribution with either the parameter’s independent effect, its interactions with other parameters or the effect of correlations among inputs. This decomposition clarifies how the model structure and the parameter landscape influence the output variability in the generated VPC, allowing us to identify whether a specific input parameter’s impact is predominantly due to its main effect, due to interactions with other inputs or is largely driven by its correlated behaviour with other parameters.

#### (ii) Calculation of the variability and sensitivity indices

The sensitivity indices and the associated variance were computed while correlations were considered, using the copula-determined correlation matrices derived in §2b(v). The accuracy level of the Smolyak algorithm was set to 4, representing the seventh-order polynomial exactness. We examined the convergence of the indices for k=2 , 3 and 4 . The relative contributions of the relevant parameters when using k=4 showed minimal changes compared with k=3 , whereas significant changes were observed when compared with k=2 . Specifically, the maximum absolute difference between k=2 and k=3 was found to be 0.34. In contrast, the maximum absolute difference between k=3 and k=4 was 0.11. We found that the most important features remained similar for each analysis, and only small quantitative differences were found for various values of *k*. Given that extending the analysis to k=5 was found to be computationally infeasible, we have therefore chosen to proceed with all subsequent analyses using k=4 . We provided the indices for the different *k*-values for patient-2L FFR LAD in electronic supplementary material, appendix F. Uniform input distributions were assumed, resulting in the inputs of the SA being the mean vector and the derived correlation matrix. The obtained outputs were the expected value, the output variance and the sensitivity indices belonging to the model inputs.

## 3. Virtual patient cohort generation

Upon validation of the one-dimensional pulse wave propagation model for its efficacy in simulating a generic patient (see electronic supplementary material, appendix A), the model was subsequently used within a VCG framework to develop a VPC by employing the random input variation and output filter methodology.

### (a) Haemodynamic heterogeneity within the generated virtual patient cohort

**Figure 4.**
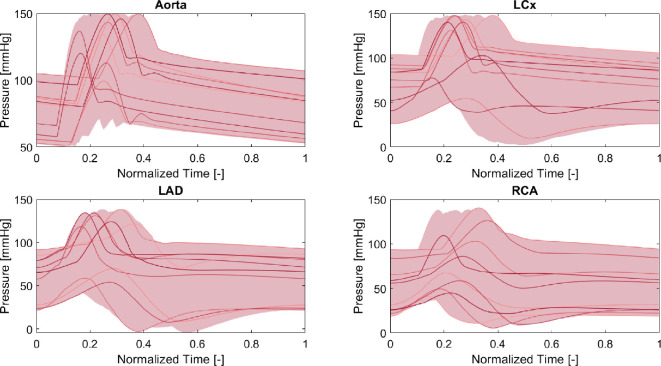
Variability in pressure waveforms of the generated VPC. The shaded area highlights the total coverage of all waveforms. The highest layer represents the upper bounds, while the lowest layer represents the lower bounds per time step. Additionally, 10 randomly chosen waveforms are illustrated to showcase inter-waveform variability.

**Figure 5.**
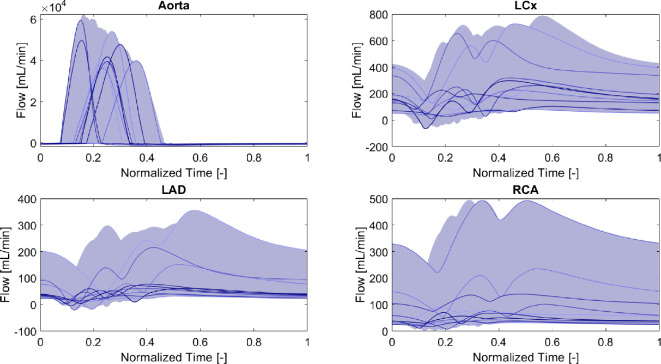
Variability in flow waveforms of the generated VPC. The shaded area highlights the total coverage of all waveforms. The highest layer represents the upper bounds, while the lowest layer represents the lower bounds per time step. Additionally, 10 randomly chosen waveforms are illustrated to showcase inter-waveform variability.

Figures 4 and 5 show the aortic haemodynamics present within the generated VPC, obtained from the element right after the one-fibre heart model and the coronary haemodynamics present within the generated VPC. The haemodynamics of the main coronary arteries were taken from the elements right after the zero-dimensional stenosis element.

As can be seen in figure 4, coronary pressure waveforms exhibit discernible systolic peaks, while figure 5 shows that flow waveforms reveal marked diastolic peaks among most VPC participants. Aortic pressure and flow waveforms show distinct systolic peaks, and the ranges are confirmed to be within physiological bounds (maximal pressure of 150 mmHg and minimal pressure of 50 mmHg; maximal flow of 62 × 10^3^ ml min^−1^ and minimal flow of −19 × 10^2^ ml min^−1^) largely due to strict filtering criteria. The same holds for the cardiac output resulting in it being within physiological bounds as well. With regard to the coronary arteries, the pressures and flows of the LCx (maximal pressure of 149 mmHg and minimal pressure of 2 mmHg; maximal flow of 789 ml min^−1^ and minimal flow of −64 ml min^−1^) and RCA (maximal pressure of 140 mmHg and minimal pressure of 5 mmHg; maximal flow of 495 ml min^−1^ and minimal flow of ml min^−1^) meet acceptable limits [38,40]. However, examining the LAD (maximal pressure of 139 mmHg and minimal pressure of −5 mmHg; maximal flow of 356 ml min^−1^ and minimal flow of −20 ml min^−1^), although the pressures remain largely physiological, some virtual patients exhibit negative pressures just after the zero-dimensional stenosis element. The negative pressure could possibly be attributed to a negative wave reflection, but further investigation is warranted. Comparatively, flow waveform ranges in the LAD in our study were lower than those reported by Fournier *et al*. [40]. However, their subjects had no or only mild coronary artery disease. Our focus pertained to multi-vessel disease patients from the FAME study, justifying the observed reductions and variations in flows. As for the haemodynamics, the filtering strategy ensured a representative cohort. Nonetheless, additional filtering might optimize the cohort, especially with regard to the hyperaemia factor since it is related to the severity of the stenosis [36], which was currently not taken into account. A good additional filtering approach could be to ensure that the hyperaemia factor is applied per vessel and matches the severity of the stenosis, inducing a strong correlation between both input parameters.

### (b) Agreement of virtual patient cohort with real patient cohort

**Figure 6.**
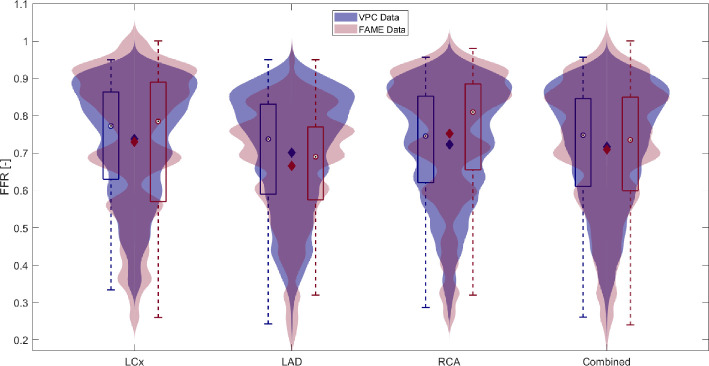
Violin plots representing the generated VPC and the FAME study and their distribution in predicted FFR values per stenotic lesion. The blue violin and box plots belong to the VPC, whereas the red plots belong to the data of the FAME study. The diamonds represent the mean, whereas the dot within the box plot is the median.

The FFR distribution in the generated VPC and FAME study can be seen in figure 6. The subset of 167 patients from the FAME study was chosen based on the presence of FFR measurements exclusively within the main coronary arteries (LCx, LAD and RCA) and on no lesions present in consecutive segments and around bifurcations. The mean of the FFR values found within the LCx in the VPC and FAME study was 0.74 and 0.73, respectively. For the LAD, for the VPC and the FAME study the means were 0.70 and 0.67, respectively. The RCA was found to have a mean FFR of 0.72 and 0.75 for the VPC and FAME study, respectively. Overall, the mean FFR was found to be 0.72 and 0.71 within the VPC and the subset of the FAME study, respectively. The distributions are slightly skewed to the left. Notably, the VPC distribution appeared more smooth compared with the FAME subset. This difference can be attributed to the larger sample size in the VPC (patient-2L: 771; patient-3L: 772 FFR values) versus the FAME study (LAD: 132, LCx: 82, RCA: 100 FFR values). Despite the difference in distribution shapes, box plots revealed that medians, interquartile ranges and the range between maximum and minimum values were in good agreement between the two datasets.

Table 3 shows the results of the KS test, which was applied to FFR values between the FAME and VPC datasets. For the overall comparison of the distributions, the KS statistic (0.07) shows minimal differences that are not statistically different (*p* = 0.11). However, the KS statistics for the individual vessels show some slight differences for the LAD (KS-test statistic = 0.2, *p* = 0.0003) and the RCA (KS-test statistic = 0.16, *p* = 0.01) but not for the LCx (KS-test statistic = 0.15, *p* = 0.13).

**Table 3.** Kolmogorov–Smirnov test results for FAME versus VPC data.

vessel	KS statistic	*p*‐value
LCx	0.1370	0.1159
LAD	0.1884	0.0003
RCA	0.1613	0.0133
combined	0.0703	0.1095

It is evident from figure 6 and table 3 that overall the distributions and means of the VPC and the subset of the FAME data agree relatively well. Further optimization of the distributional agreement could be achieved by additional filtering. SA might aid in providing a structured approach for designing the filtering strategy.

#### (i) Future virtual patient cohort applications

In future applications, once the VPC has been properly validated for specific purposes, it could be utilized in various scenarios. For instance, it could be employed to assess the number of stents placed based on the stenosis severity (where stenting occurs if severity>50% ). Subsequently, this process could be repeated based on the evaluation of the computed FFR (where stenting occurs if FFR<0.8 ) within the same population. If the average number of stenting procedures closely matches that of the FAME study, which also had these two experimental groups, we will have effectively validated our cohort for its suitability in an ISCT to reproduce the findings of the FAME study. Furthermore, the VCG could later be expanded to deal with possible coronary interventions such as percutaneous coronary intervention and coronary bypass graft surgery. This allows for the creation of a VPC that can be utilized to explore different intervention methods in various ways, i.e. optimal stenting strategies. Possibly, these virtual patients could later on be used as templates for patient-specific modelling. These models could, for instance, be used to derive a patient-specific threshold value with regard to FFR measurements, as opposed to the universally applied 0.8 threshold value.

## 4. Surrogate model construction for efficient sensitivity analysis

To efficiently derive the input–output relationship of the VPC, surrogate models were created to reduce the computational costs of the SA greatly. For patient-2L, two surrogate models were created: one for the FFR of the stenosis present in the LAD and one for the FFR of the stenosis present in the RCA. For patient-3L, three surrogates were generated for the FFR in each main coronary artery. To evaluate the performance of the surrogates, the training set was evaluated and the surrogates were validated using an independent test set.

### (a) Performance of the surrogate models

**Figure 7.**
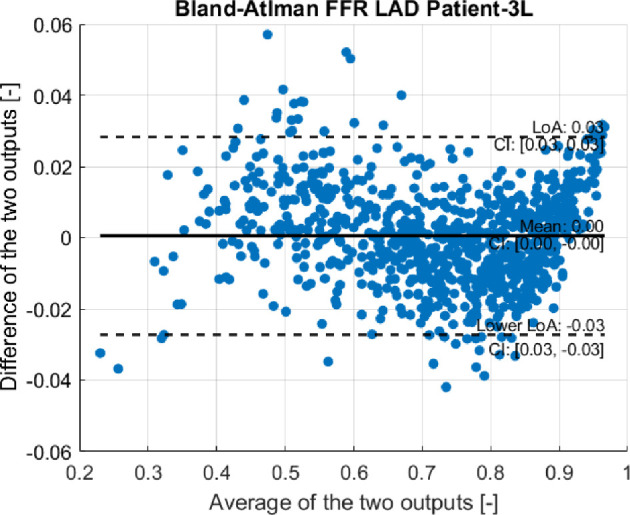
Bland–Altman plot of the surrogate model of the LAD FFR of patient-3L.

The Bland–Altman plot in figure 7 reveals a mean difference (represented by the thick black line) of $5.38\times 10^{-4}$ (-) for the FFR of the LAD for patient-3L, which indicates a small discrepancy between the outputs of the test set and the predictions of the surrogate model. The pattern and statistics found in this Bland–Altman are indicative for all other surrogates; hence we only provided the Bland–Altman plot for the surrogate of the FFR LAD for Patient-3L. For all surrogates, the absolute mean difference was on average $2.69\times 10^{-4}$ (-). For each surrogate model, FFR values below 0.6 tend to be overestimated, with an average upper limit of agreement (LoA) of LoA=0.03 (as indicated by the upper dotted line). The upper LoA represents the upper boundary within which 95% of the differences between the surrogate and test set results are expected to fall. Conversely, for values above 0.6 , a slight underestimation is observed, with the lower limit of agreement averaging lowerLoA=−0.03 (as shown by the lower dotted line). The lower LoA defines the corresponding lower boundary for these differences. This pattern is consistent across all surrogate models.

Table 4 confirms the performance metrics for each surrogate model, with a maximum normalized RMSE of 0.02 (-) for the surrogate of the LAD FFR of patient-3L. Despite a large input space and complex input–output dynamics, the surrogates demonstrated excellent performance. Their performance surpasses the typical accuracy of FFR measurements, recognized for their high reproducibility and low variability, indicated by a standard deviation of 0.018 and a coefficient of variation of 2.5% [46]. This accuracy validates the applicability of the surrogate models in SSA.

**Table 4.** Root mean square error (RMSE) and normalized root mean square error (nRMSE) for the FFR surrogate models.

model	$\epsilon_{RMSE}$ (-)	$\epsilon_{nRMSE}$ (-)
model patient 2L FFR LAD	$1.33\times 10^{-2}$	$1.83\times 10^{-2}$
model patient 2L FFR RCA	$1.36\times 10^{-2}$	$1.79\times 10^{-2}$
model patient 3L FFR LAD	$1.39\times 10^{-2}$	$1.93\times 10^{-2}$
model patient 3L FFR LCx	$1.29\times 10^{-2}$	$1.69\times 10^{-2}$
model patient 3L FFR RCA	$1.40\times 10^{-2}$	$1.85\times 10^{-2}$

### (b) Limitations of the surrogate models

**Figure 8.**
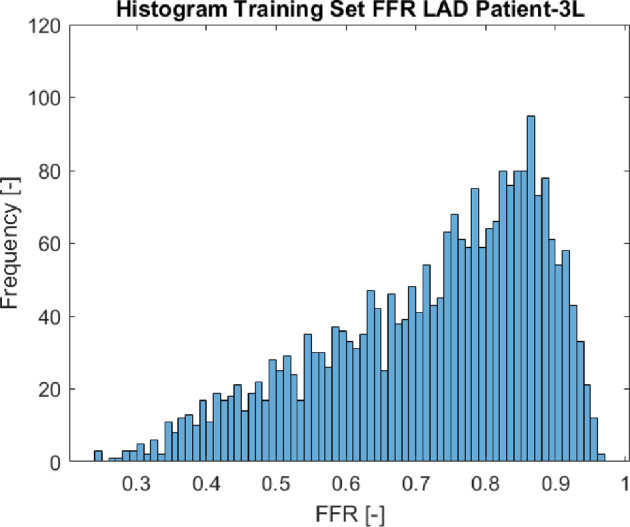
Histogram of the training set of the LAD FFR of patient-3L.

As observed in the Bland–Altman plot illustrated in figure 7, the FFR surrogates exhibited a tendency to both overestimate and underestimate the FFR values across various FFR regions. This behaviour could be attributed to the asymmetry observed in our outputs of interest within the training set, as demonstrated in the histogram of figure 8. The smallest error was noticed within the Bland–Altman plots between an FFR of 0.7 and 0.9, which is the most data-dense region, as can be seen in the histogram of figure 8. However, when the surrogates had to make predictions from the less data-dense regions for FFR values below 0.7, they tended to exhibit over- and under-prediction. To address this issue, we experimented with various kernels such as Gaussian, IMQ and Wenland RBF kernels to determine if they could better fit the data and prevent this pattern. However, none of them resulted in a linear relationship around the zero axis of the Bland–Altman plot. The asymmetry in the training data could be causing this distinct wave shape within the Bland–Altman plots, which warrants further investigation.

It is possible that enhancing the uniformity of the output space by including more data points in the lower regions of the FFR values could improve the performance of the surrogate models. Adding more data points, in turn, will also result in slightly better surrogate models, as shown in [15].

## 5. Exploring the variability and parameter landscape of the generated virtual patient cohort

Variance-based SA was utilized to explain the variability in FFR resulting from the VCG for *in silico* clinical testing and to pinpoint which input variables of the VCG, including their possible correlations and interactions, significantly impact the variability in the predicted FFR values belonging to the VPC and, thus, affect the spread within the VPC.

### (a) Fractional flow reserve variability of the virtual patient cohort

Initially, the variance for each output of interest was calculated for each type of patient. Table 5 presents the calculated expected values alongside their respective variances. It can be inferred that the average expected value of the FFR is 0.74 (-), with an average variance of 0.02 (-). Moreover, the square root of the variance is determined to be 0.14 (-). These computed statistics closely align with those found in the data of the FAME study [13]. Specifically, the computed expected FFR values for LAD, RCA and LCx are 9, 1 and 5% higher, respectively, with the combined expected value being 7% higher. Additionally, variances and the square root of the variances demonstrate similar consistency, underscoring the credibility of our findings. The relatively high square roots of the variances indicate substantial variability in FFR values within the VPC simulations and FAME study, which could also be seen in figure 6. Significant variability is not necessarily a flaw in the dataset or simulations; rather, with respect to (virtual) patient cohorts, it reflects the intrinsic variability of FFR measurements between (virtual) patients with coronary artery disease.

**Table 5.** The expected value, variance and the square root of the variance found for each output of interest and the corresponding statistics from the subset of the FAME study [13].

output of interest	expected value	variance	$\sqrt{V(Y)}$ (-)
	E(Y) (-)	V(Y) (-)	
patient-2L FFR LAD	0.72	0.02	0.15
patient-3L FFR LAD	0.72	0.02	0.15
FAME statistics LAD	0.67	0.02	0.16
patient-2L FFR RCA	0.75	0.02	0.14
patient-3L FFR RCA	0.75	0.02	0.14
FAME statistics RCA	0.75	0.03	0.18
patient−-3L FFR LCx	0.76	0.02	0.13
FAME statistics LCx	0.73	0.04	0.19

### (b) Sensitivity analysis

**Figure 9.**
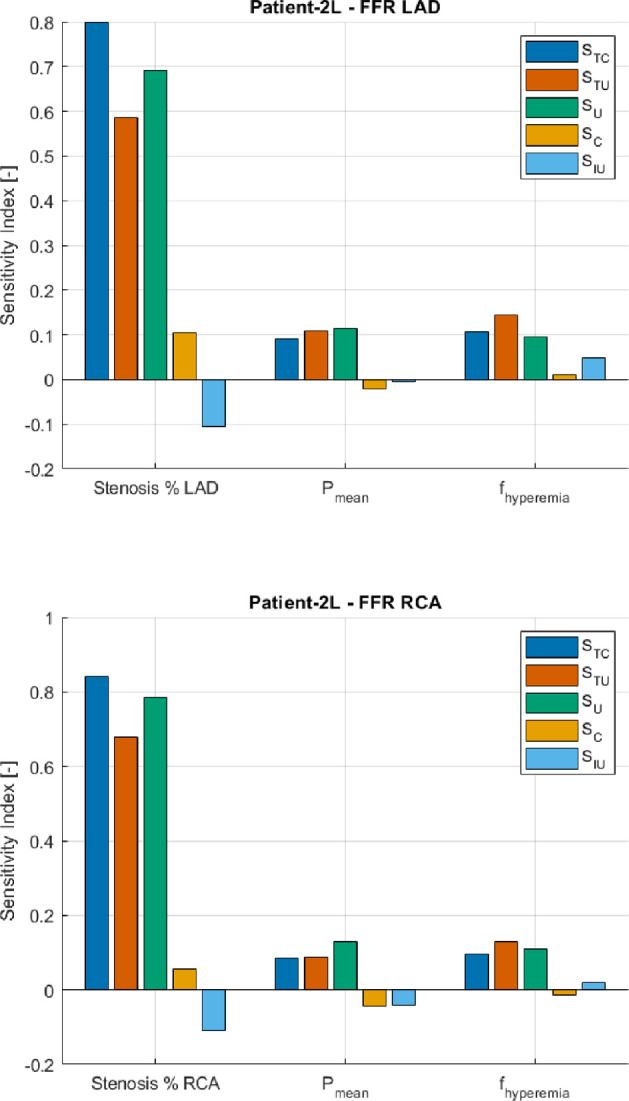
Correlated sensitivity indices for patient-2L.

**Figure 10.**
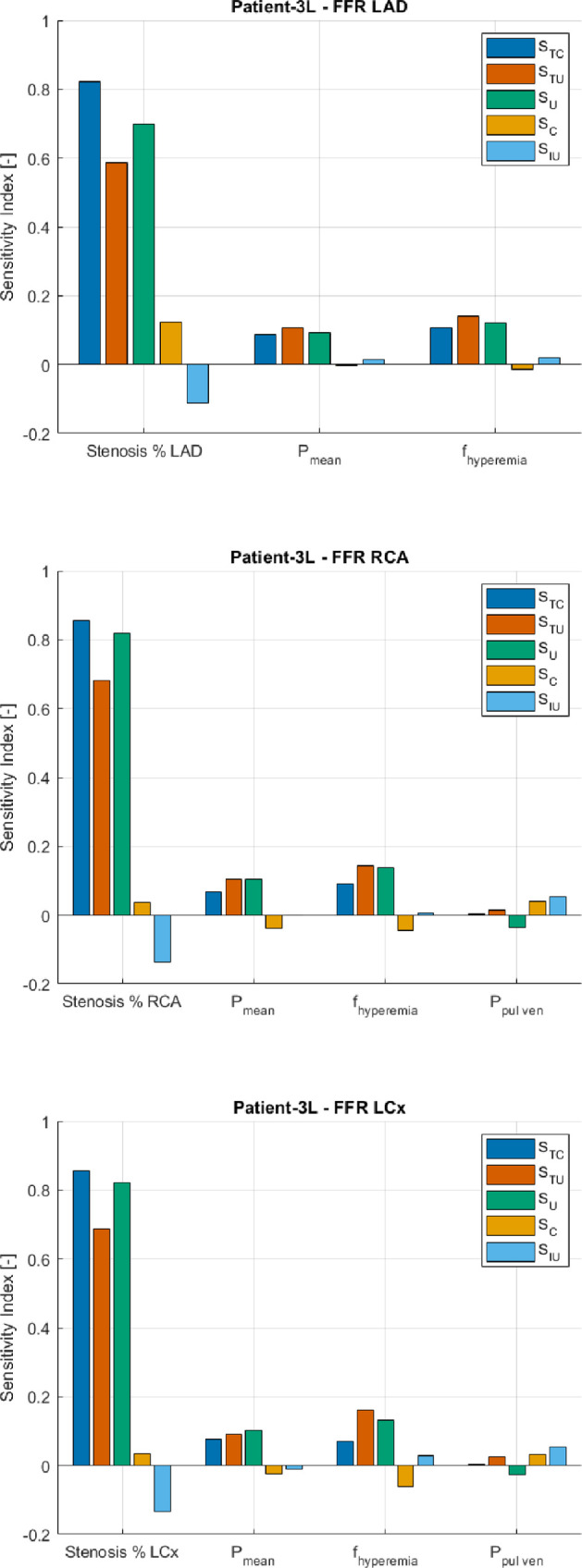
Correlated sensitivity indices for patient-3L.

To explain the computed variability, we turn to the resulting sensitivity indices from the SA. Figures 9 and 10 show the correlated sensitivity indices, as defined in §2d, of the input parameters of the VCG. Only the indices were shown for the inputs with at least one index higher than 0.05 (-). Conversely, all other inputs have sensitivity indices lower than 0.05 (-). From both figures 9 and 10, it can be derived that the stenosis percentage of each main coronary artery is a key driver of the variability of the VPC. If we take, for instance, the stenosis percentage of the LAD (stenosis % LAD) for patient-2L, it can be seen that the stenosis percentage contributes significantly to the variability on its own, indicated by an $S_{U}$ of 0.69 (-). However, this stenosis percentage LAD of patient-2L is also somewhat involved in correlations and interactions with other input parameters, indicated by an $S_{C}$ and $S_{IU}$ of 0.11 (-) and −0.11 (-), respectively. From the negative $S_{IU}$ , we can thus derive that interactions of the stenosis percentage LAD for patient-2L with other inputs contribute slightly negatively to the variability within the VPC. Similar observations can be seen for the stenosis percentages in the other main coronary arteries.

The mean pressure ( $P_{mean}$ ) and the hyperaemia factor ( $f_{hyperaemia}$ ), for each stenotic lesion, are also key drivers of the variability in the VPC. Similar to the aforementioned input, the contribution to the variability of the VPC is mainly due to the parameters themselves. Still, they are also slightly involved in positive and negative correlations. It should be noted that all these contributions are much smaller than the contribution due to the parameter itself ( $S_{U}$ ). For Patient-3L, the interactions between the pulmonary venous pressure (*P*_*pul ven*_) and other input parameters contribute slightly and positively to the variability of the VPC for stenoses in the RCA and LCx, as indicated by an S_IU_ value of 0.05 [−]. However, the pulmonary venous pressure is not identified as a key driver of variability because the other sensitivity indices exhibit only minor effects. Additionally, we provided all correlated sensitivity indices for each model input, which can be found in electronic supplementary material, appendix G.

### (c) Convergence sensitivity indices

When performing SA, it is important to check whether or not the indices converge. In this study, the Smolyak algorithm was configured to an accuracy level of 4, corresponding to the seventh-order polynomial exactness. This configuration resulted in a grid comprising 54 877 points for patient-2L and 64 969 points for patient-3L. However, this level of accuracy might not have achieved exact convergence, as the importance ranking of the sensitivity index due to interactions ( $S_{IU}$ ) for the hyperaemia factor continued to change, as demonstrated in electronic supplementary material, appendix F. Furthermore, with 34/36 input parameters, we estimated that performing the SA at k=5 would require up to $1.3\times 10^{6}$ CPU hours and 83 days of execution time, utilizing 184 GB of memory and 20 cores. Consequently, conducting the SA becomes excessively intensive computationally due to the curse of dimensionality, even when using surrogate models. Nonetheless, the overall conclusions of the SA remain unchanged, and the differences between sensitivity indices at k=3 and k=4 are significantly smaller than those between k=2 and k=3 , suggesting that the results might have converged.

Still, to adequately assess whether our sensitivity indices have fully converged using *k* = 4, it would be necessary to perform the SA with an even larger number of grid points. To tackle the curse of dimensionality and reliably assess the convergence of the sensitivity indices, one strategy is to reduce the dimensionality of the input space by eliminating certain inputs, thereby allowing the Smolyak algorithm to achieve higher accuracy. For this, a screening method of Morris could be applied prior to the SA [47], or one could omit certain input parameters that are considered well established from the SA altogether. However, it is important to note that sensitivity indices only reflect an input’s contribution to the variance relative to the other inputs being analysed. Therefore, modellers should exercise caution when deciding which input parameters to disregard in their SA. Additionally, in the context of biomedical models, input spaces often have very high dimensionality, potentially making it impractical to reduce the input space to a desirable level. Future work should focus on developing more efficient algorithms and techniques that can handle high-dimensional input spaces while maintaining extensive grid point sets via the implementation of the Smolyak algorithm. Other work could focus on effective screening methods to safely reduce the input parameter dimensions of the SA.

## 6. General discussion

In this study, we explored the input–output relations and the parameter landscape of a VPC, comprising a physiological model and its filtered input–output pairs. To this end, a VPC was created using a one-dimensional pulse wave propagation model of the coronary circulation as part of a VCG by filtering out non-physiological outputs, possibly introducing correlations. Surrogate model-based SA, capable of accounting for these correlations, was utilized to identify the variability of the predicted FFR and the critical drivers of the variability within the VPC. With the VCG, we could recreate the FAME study population on a global level, i.e. predicting the average FFR values per main coronary artery.

### (a) Sensitivity analysis for examining the variability and parameter landscape in virtual patient cohorts

Our variance-based SA approach allowed us to identify key drivers of the derived variability within our synthetically generated VPC. Moreover, the decomposition of the $S_{TC}$ and $S_{TU}$ into the $S_{C}$ , $S_{U}$ and $S_{IU}$ provided the modeller with more insight into the output distribution of the VPC, the underlying model structure of the VCG and the parameter landscape of the VPC; whether the variability could be mainly explained due to the parameter on its own, or due to correlation and/or interaction between the parameter and other parameters. These results can help us more accurately define the proper input ranges, resulting in the correct variability within the output. When the key drivers’ input ranges are optimized, these input ranges become essentially fixed. As a result, another input parameter (range) could then become a key driver, even though it was not as important relative to the previously discerned key drivers. This shift occurs because the SA reallocates the contribution to the variability among the remaining uncertain parameters. This essentially captures the essence of SA for virtual cohort validation: a process that should be repeated at various stages of the modelling exercise for VPC generation. It helps the modeller validate their VPC on a population level and understand the input–output relationship of the model itself.

During cohort validation on a population level, the modeller assesses whether the variability within the cohort is either too great or too small [6]. The SA can be applied to derive the key drivers, which is crucial for generating a heterogeneous set of virtual patients that mimics the variability observed in the real-world population [5]. For instance, in the case where there is less variability within the cohort than in the real population, which is often an issue possibly due to an underestimation of the variability of specific input parameters [6], SA can help the modeller find the input parameter that perhaps requires a larger sampling range. Moreover, when the contribution is mainly found to be due to the correlations between that input parameter and other parameters, it might be wise to try to find out what the underlying correlation structure is and how it affects the output of interest. Another virtual cohort validation method is by comparing the differences in the cumulative distributions [6]. Based on the KS-test, we found that the synthetic VPC of this article, overall, showed no significant differences in the shape of the distribution when compared with the FAME study data. However, we did find statistical differences for the individual vessels, namely the LAD and RCA. Hence, correlated SA needs to be applied to understand the model structure and parameter landscape better and improve these distributions. However, drawing conclusions with regard to how parameters affect the distribution shape is much more challenging than changing the overall variability of the VPC. Finally, there could also be differences in the output distributions due to missing mechanisms within the model structure [6]. However, future research needs to determine whether this method can be used to identify the key factors driving these differences.

### (b) Improving the virtual cohort generator and the resulting virtual patient cohort

The current SA tells us the variability of the FFR of the VPC population is mainly attributed to the severity percentage of the stenosis (stenosis %) for each of the outputs of interest in this study. This also suggests that regardless of the location of the stenosis within a segment, the degree of radius reduction has the most significant impact on the FFR distribution. Consequently, using a general template geometry to represent different patients seems to be a promising approach for gradually moving towards a robust VCG for ISCTs, but more verification is needed. A potential next step would be also to explore how effective the use of a general template geometry would be in a clinical setting.

With the severity percentage of the stenosis being a key driver in the variability of the VPC, our focus should turn towards the zero-dimensional stenosis model, which is overly simplified and idealized, presupposing that all stenotic lesions exhibit perfect axis-symmetric concentric characteristics. This model variable adjusts the sinusoidal shape of the stenosis solely based on the length and severity of the stenotic lesion as denoted in the literature [35]. Contrarily, empirical evidence presents stenotic lesions as being considerably more complex, ranging from eccentric to diffuse in nature [48]. Such geometric intricacies have been demonstrated to significantly affect coronary haemodynamics and the resultant FFR value [49,50]. The current stenotic lesion model within the VCG cannot address these complexities and, therefore, does not perfectly capture the proper variability within the VPC. Due to the profound influence of the severity percentage of the stenosis on the variability of the FFR within the VPC, it may be worthwhile to consider refining the existing stenosis model. In this manner, the severity percentage of the stenosis can be more specifically defined, possibly leading to an even more realistic shape in the FFR distribution within the VPC. It should be noted, however, that often it is complicated, costly, and time-consuming to obtain an adequate three-dimensional representation of a stenotic lesion.

Regarding the mean pressure being identified as another key driver of the variability within the VPC, the recommendation leans towards clearly defining the input sampling range based on measurements, given its relative simplicity and accuracy in measurement. As for the hyperaemia factor, the model should be improved to allow for the hyperaemic condition to not only be applied uniformly across all microvascular resistances but rather to be adjusted per vessel depending on the stenosis degree [36]. Both the mean pressure and the hyperaemia factor contribute to the calculation of total coronary resistance, which directly influences the coronary flow and, consequently, the FFR.

### (c) Future recommendations

In the case of using the data from the FAME study, creating an exact virtual cohort, for instance, through 1:1 mapping, is not feasible due to the sparsity of the dataset and disagreement between that dataset and the model’s input space. Achieving a patient-specific model via 1:1 mapping would require a complete patient-specific dataset (both boundary conditions and geometry), whereas the current approach manages with only anatomical information and generic boundary conditions. Additionally, it is essential to acknowledge that conducting an RCT twice does not yield identical results each time, highlighting the inherent variability in real-world outcomes. Therefore, striving for a 1:1 replication of a population is not only an impractical goal but also an unnecessary one. The value of a VPC lies in its ability to be representative of a population with reasonable accuracy, thus offering significant utility in gaining specific insights without the need for exact replication of an RCT. In the future, performing SA in this manner onto a VPC will help provide insight into the spread of the output of the VPC and the input–output relationship of the VPC.

As the virtual cohort within this study was able to capture the population spread found within the FAME study relatively well, the VCG could perhaps, in the future, be used to create VPCs of scenarios before and after stenting. Hence, using SA, more can be learned about the input–output relationship of those VPCs, which in turn can guide necessary adjustments to better align the VPC with actual data. Once validated, the VCG may then serve to generate VPCs with greater homogeneity or alternative outputs of interest that can be used for *in silico* testing. These, in turn, can then be used for *in silico* testing on certain specific subgroups of a cohort. The SA approach presented in this article is not only beneficial for our current application but is also recommended as a general strategy whenever VPCs are constructed in other modelling contexts.

## 7. Conclusion

This study has successfully demonstrated a methodology for performing variance-based SA on a VPC with a considerably large input space. This was done while considering the possible induced correlations between the inputs, which could be introduced when generating a VPC through random sampling and filtering within physiological bounds. Although not without its limitations, this SA approach represents an effective method for evaluating a VCG and the resulting VPC. Additionally, it was found that the severity percentage of the stenosis influenced the variance within the FFR values of the VPC the most. Future research may uncover more optimized techniques for handling complex models, such as the pulse wave propagation model and large input spaces. Nonetheless, this work marks a significant step forward in our ability to validate and enhance the one-dimensional pulse wave propagation model as part of a VCG for FFR predictions in *in silico* clinical testing.

## Data Availability

The model-generated VPC data along with the code used to process and generate all figures are available in our repository on Zenodo [51]. The FAME data, the one-dimensional pulse wave propagation code and the sensitivity analysis code remain available upon reasonable request. For access to these codes, please contact the corresponding author, Professor Wouter Huberts; inquiries related to the FAME data will be directed to Professor Tonino, the dataset's owner. Supplementary material is available online [52].
